# Accumulation of Heavy Metals from Battery Waste in Topsoil, Surface Water, and Garden Grown Maize at Omilende Area, Olodo, Nigeria

**DOI:** 10.1002/gch2.201700090

**Published:** 2018-02-05

**Authors:** Adedotun Onoyinka Afolayan

**Affiliations:** ^1^ Tissue Culture/Biotechnology Unit National Centre for Genetic Resources and Biotechnology P.M.B. 5382, Moor Plantation Ibadan Oyo State +234‐02 Nigeria

**Keywords:** cadmium, heavy metals, lead, maize, waste dumpsite

## Abstract

Land pollution is a threat to sustainable agricultural development and food security in developing countries. Consumption of farm products from contaminated areas can generate health hazards to the diverse consumers along the food chain through the different pollutants in the products. This study is designed to determine the accumulation of Pb, Cd, and Fe in topsoil, surface water, and maize leaf, stem, grains, and root, cultivated in a garden nearby Ori‐Ile battery waste dumpsite, Omilende Area, Olodo, Nigeria. Soil samples, garden maize parts, and surface water samples are collected from the study area using standard procedures. Corresponding reference samples are collected from Moor Plantation, Ibadan. All collected samples are analysed for Pb, Cd, and Fe concentrations. Mean Pb, Cd, and Fe concentrations in topsoil are found to be significantly higher than 157.0 ± 39.8, 2.2 ± 1.2, and 976.3 ± 353.9 mg kg^−1^, respectively, which are obtained from reference soil and National Environmental Standards and Regulations Enforcement Agency limits (Pb: 164 mg kg^−1^ and Cd: 50 mg kg^−1^). The soil contamination factor values obtained are greater than 6, indicating severe pollution. Downstream has the highest Pb, Cd, and Fe concentrations. In maize parts, the root has the highest concentration of Pb (40.95 ± 1.98 mg L^−1^) and Cd (2.84 ± 0.19 mg L^−1^), which are significantly higher (*p* ≤ 0.05) than those from the reference site. A high concentration of heavy metals found in topsoil further bio‐accumulates in maize parts. Consequently, this garden maize is unfit for consumption.

## 1 Introduction

Uncontrolled open dumping on the peripheries of many cities has resulted in the degradation of valuable land resources and the creation of long‐term environmental and human health problems.^1^ Land contamination and degradation are a threat to sustainable agricultural development and food security in developing countries.^2^ Numerous industrial activities, including automobile battery production, have often resulted in the accumulation of noxious metals in the environment,^3^, ^4^, ^5^, ^6^, ^7^ and the discharge of heavy metals as a by‐product of these activities has been accompanied by large‐scale soil pollution.^7^, ^8^ Heavy metals tend to persist in the environment indefinitely^9^ and get accumulated over time in soils, water sediments, and plants. Hence, they could have a negative influence on physiological activities of plants, determining the reductions in plant growth, dry matter accumulation, and yield.^10^ Excessive accumulation of heavy metals in agricultural soils often leads to elevated heavy metal uptake by crops, and thus affects food quality and safety.^11^ Food chain contamination is one of the important pathways for the entry of toxic pollutants into the human body,^12^ and the consumption of heavy‐metal‐contaminated food can seriously deplete some essential nutrients in the body which are further responsible for decreasing immunological defenses, intrauterine growth retardation, impaired psychosocial faculties, disabilities associated with malnutrition, and high prevalence of upper gastrointestinal cancer rates.^13^, ^14^ Heavy metal accumulation in plants depends upon plant species, and the efficiency of different plants in absorbing metals is evaluated by either plant uptake or soil‐to‐plant transfer factors (TFs) of the metals.^15^ Some of the heavy metals such as Pb and Cd are toxic to plants and animals, even in trace concentrations,^16^ and exposure to some of them is normally chronic due to food chain transfer.^17^ Pb and Cd have been found to inhibit plant growth, disturb ion uptake and transport, as well as inhibit enzyme activation and photosynthesis.^18^, ^19^ Fe is essential for many plant functions^20^ but is toxic when it accumulates to high levels. Excess Fe can result in dark green foliage, stunted growth of tops and roots, dark brown to purple leaves in some plants.^20^ Emissions of heavy metals to the environment occur via a wide range of processes and pathways, including air, surface water, and soil, and subsequently, ground water and crops.^21^ Surface water can be polluted by contaminants that are washed into it from a nearby polluted site.^21^

Remediation of soil contamination by conventional engineering techniques is often a very expensive procedure. This is often the reason why the cleanup of contaminated areas has not been taking place at a first level. However, bioremediation techniques, especially those that involve the use of metal hyperaccumulating plant species, are well known and adopted remediation alternatives nowadays. Certain plants can flourish in soil polluted to levels that are often orders of magnitude higher than current regulatory limits, which are often set relatively independent of plants' tolerance limits and are most often derived from human health and aquatic toxicology end points.^22^ These metal hyperaccumulating plants have a great diversity of genetic adaptations, which they employ in handling potentially toxic levels of metals and other pollutants that occur in the environment. Phytoremediation takes advantage of plants' nutrient utilization processes to take in water and nutrients through roots, transpire water through leaves, and act as a transformation system to metabolize organic compounds, such as oil and pesticides. Alternately, they may absorb and bioaccumulate toxic trace elements including the heavy metals.^23^ One of such crops is maize or corn. It is a cereal crop with fast‐growing property and hyperaccumulating ability.

Omilende suburb is part of Ikumapaiyi area of Olodo, Ibadan, Nigeria, and it has a portion that has been named “Ori‐Ile,” which is one of the several areas used as open dump by the waste contractors of the now closed‐down lead acid battery manufacturing company. The allocation of these previously used open waste dumps for housing development has presented a series of environmental challenges to the inhabitants.^24^ The residents of Ori‐Ile Olodo predominantly grow maize for both human and animal consumption while some of them utilize their harvested maize seeds for constituting feed for their domestic animals.^21^ Maize is an important staple crop^25^ consumed by the population of Nigeria in large amount.^26^ Maize ranks third in the world production of cereal following wheat and rice.^27^ It is used as feed for livestock and a principal raw material for many industrial products.^26^, ^27^ Maize, classified as *Zea mays*, is the common name for the cereal grass in the Poaceae, which is known to be a good accumulator of contaminants.^28^ The uptake, metabolism, and negative effects of heavy metals on different plants including maize have been documented in literature.^29^, ^30^ However, the mechanism of accumulation of these heavy metals is still not completely understood^29^ especially since Pb and Cd are the most widespread no‐nutrient heavy metals.^31^While the study conducted by Olusoga and Osibanjo^23^ at Olodo Ibadan touched on the concentration of lead and cadmium in the whole maize plants, the present study is designed to determine the accumulation of three selected heavy metals, namely lead (Pb), cadmium (Cd), and iron (Fe) in topsoil, surface water, and maize grown in gardens around Ori‐Ile battery waste dumpsite, Omilende Area, Olodo, Ibadan Nigeria, by analyzing their concentration within the topsoil and surface water samples alongside the selected maize parts namely leaf, stem, grains, and root.

## 2 Results and Discussion

### 2.1 Pb, Cd, and Fe Concentrations in Soil Samples

**Figure 1:**
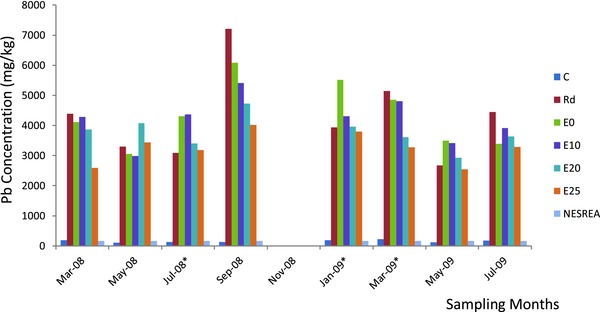
Bimonthly concentration of lead in soils of waste dumpsite (Rd) and along experimental garden direction. Key: *, significant; each bar, sampling months; successive colors, collection points' lead content (C: reference soil, Rd: waste dumpsite soil, and E: experimental garden direction soil).

**Figure 2:**
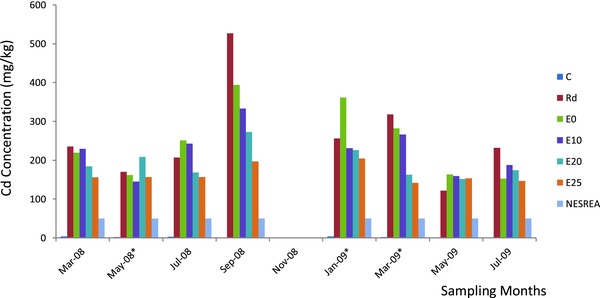
Bimonthly concentration of cadmium in soils of waste dumpsite (Rd) and along experimental garden direction. Key: *, significant; each bar, sampling months; successive colors, collection points' cadmium content (C: reference soil, Rd: waste dumpsite soil, E: experimental garden direction soil).

**Figure 3:**
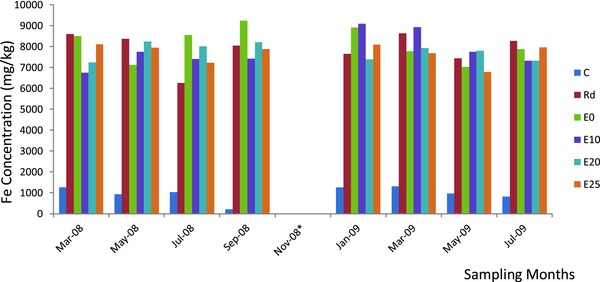
Bimonthly concentration of iron in soils of waste dumpsite (Rd) and along experimental garden direction. Key: *, significant; each bar, sampling months; successive colors, collection points' iron content (C: reference soil, Rd: waste dumpsite soil, E: experimental garden direction soil).

In the topsoil samples collected from the waste dumpsite (Rd sample) and the distances sampled along the experimental garden direction from the edge of the waste dumpsite (E), a wide range of soil Pb, Cd, and Fe concentrations were observed (**Table** **1**; **Figures** **1**–**3**). In Rd and E soils, Pb, Cd, and Fe concentrations were significantly higher (*p* ≤ 0.05) compared with the reference soils (C). The results indicated that Pb and Cd concentrations exceeded the Environmental Quality Standards set by National Environmental Standards and Regulations Enforcement Agency (NESREA) for soils in Nigeria (Table 1). However, there was significant accumulation of Pb, Cd, and Fe in the waste dumpsite soils and those toward the experimental garden direction compared to the reference soils. The contamination factor (CF) and pollution load indices (PLI), respectively, using the reference soil concentrations of this study, were significantly very high (Rd: Pb = 27.22 and 9.45; Cd = 117.27 and 15.38; Fe = 8.10 and 6.31; E: Pb = 24.81 and 9.16; Cd = 94.32 and 14.31; Fe = 8.05 and 6.30). Overall, Rd topsoil had the highest CF. All the soil PLI values were above 1, and this further indicated significant heavy metal accumulation and pollution in soil from the study site.

**Table 1:** Mean concentrations of Pb, Cd, and Fe (mg kg^−1^) in soils of waste dumpsite (Rd) and along experimental garden direction (E). (Each value is expressed as mean ± standard deviation. Rd: Waste dumpsite soil sample; E: Experimental garden soil sample; C: Reference site soil sample; and N.A.: Not available)

Sampling point and distance	Pb [mg kg^−1^]	Cd [mg kg^−1^]	Fe [mg kg^−1^]
Rd	4273.8 ± 1436.7	258.38 ± 123.1	7910.00 ± 791.5
E0	4351.3 ± 1068.2	248.21 ± 92.7	8130.00 ± 808.4
E10	4186.7 ± 762.0	224.29 ± 60.8	7805.41 ± 808.7
E20	3775.8 ± 527.8	193.62 ± 40.4	7769.59 ± 398.4
E25	3265.8 ± 517.8	163.96 ± 23.2	7712.90 ± 473.8
C	157.0 ± 39.8	2.21 ± 1.2	976.3 ± 353.9
NESREA	164	50	N.A.

This study has revealed that Pb, Cd, and Fe concentrations in the topsoil of the waste dumpsite (Rd) and those of distances 0–25 m along the experimental garden direction (E) from the edge of the waste dumpsite were significantly higher than the values obtained from the reference site topsoil. Also, Pb, Cd, and Fe concentrations in the Rd topsoil were significantly higher than those in the distance toward E and decreased with increased distance from Rd. Pb and Cd concentrations obtained were several folds higher than the maximum permissible limit of 164 and 50 mg kg^−1^ by NESREA.^32^

According to Oni^1^ and Chirenje et al.,^33^ Pb occurs naturally in all soils and has concentrations ranging from 1 to 200 mg kg^−1^ with a mean of 15 mg kg^−1^, but the values obtained in this study for Rd topsoil and those of distances 0–25 m along the E direction from the edge of the waste dumpsite were several times beyond this range. World Health Organization (WHO)^34^ reported that normal concentrations of Pb in soil range from 15 to 30 mg kg^−1^ but in this study Pb values were much higher than this value. Pb concentrations obtained were very high than the maximum tolerable levels proposed for agricultural soils, 90–300 mg kg^−1^.^35^, ^36^ This shows a very high level of Pb contamination in the topsoil of the study site. Onianwa and Fakayode,^37^ in their study on lead contamination of the topsoil in the vicinity of a battery factory in Nigeria, reported a mean lead level of 50–2000 mg kg^−1^. However, in this study, Pb values exceeded the range reported. In the study of Adie and Osibanjo^38^ on soil polluted by slag from an automobile battery manufacturing plant in Nigeria, a range of 243–126 000 mg kg^−1^ was reported for Pb concentration. The mean values obtained for all these samples on and within the vicinity of the dumpsite were within this range, and thus compare significantly with the result reported. A range of 419.54–10 630.04 mg kg^−1^ was reported in the studies conducted by Oyediran and Aladejana^24^ on impact assessment and safety status of the excavated waste site at Olodo, Ibadan, Nigeria. This also corroborates the values obtained in this study.

WHO^39^ reported that the median Cd concentration of soil in areas not known to be polluted ranges from 0.2 to 0.4 mg kg^−1^. The cadmium concentrations obtained in this study were far higher than these stated values. The range of 1.95–32.83 mg kg^−1^, obtained by Oyediran and Aladejana^24^ in their studies on battery waste polluted areas, was much less than the values obtained for Cd concentration in this study.

According to Eddy et al.,^40^ the background level of Fe in natural soils was stated to range widely between 3000 and 500 000 mg kg^−1^ on elemental composition of soil in some dumpsites in Nigeria. The values obtained for iron concentrations of soil in this study fall within the range reported by Eddy et al.^40^ as the background level of iron in natural soils. Oyediran and Aladejana^24^ reported the range of 32 900.08–71 250.17 mg kg^−1^ for Fe concentrations in their studies on battery waste polluted areas. The Fe concentrations obtained in this study were several folds lower than those reported.

All soil contamination factors (CF > 6) and pollution load indices (PLI > 1) were significantly high. This confirmed high pollution in the waste dumpsite and its surroundings along the direction of the experimental garden. From the contamination factor and pollution load index values obtained, Cd was the highest contaminant among all the topsoils sampled at the study site, followed by Pb and Fe. The CF and PLI values for Pb, Cd and Fe in samples collected from the waste dumpsite indicated that the topsoil on the waste dumpsite had the highest pollution, followed by the topsoil along the experimental garden directions in that order.

### 2.2 Pb, Cd, and Fe Concentrations in Surface Water Samples

The mean concentrations of Pb, Cd, and Fe in the surface water in the south gradient point of the waste dumpsite, collected from March 2008 to July 2009, were compared with the standard NESREA values and reference point (control) (**Table** **2**). Zero was taken as the concentration where the sample metal level was below the detection limit of the instrument. The results of the bimonthly analysis of surface water samples taken from three points (upstream, midstream, and downstream) showed that lead and cadmium concentrations in the upstream were 0.000 mg L^−1^ throughout the sampling months while midstream lead concentration ranged from 0.000 mg L^−1^ (March 2008 to March 2009) to 0.035 (July 2009) with a mean and standard deviation of 0.024 ± 0.002 mg L^−1^; and midstream cadmium concentration ranged from 0.000 mg L^−1^ (March–July 2008 and March–May 2009) to 0.007 (September 2008) with a mean and standard deviation of 0.002 ± 0.0003 mg L^−1^. The downstream lead concentration ranged from 0.000 mg L^−1^ (March 2008 to March 2009) to 2.150 (May 2009) with a mean and standard deviation of 0.28 ± 0.01 mg L^−1^ and downstream cadmium concentration ranged from 0.000 mg L^−1^ (March–May 2008 and March 2009) to 0.171 (May 2009) with a mean and standard deviation of 0.025 ± 0.001 mg L^−1^. The results obtained showed that the concentration of lead and cadmium in the control stream was 0.000 mg L^−1^ throughout the sampling months. Overall, downstream had the highest mean lead concentration (0.275 mg L^−1^) and mean cadmium concentration (0.025 mg L^−1^), which was significantly higher than control (*p* ≤ 0.05) and NESREA standard limits (Pb = 0.01 mg L^−1^; Cd = 0.005 mg L^−1^), while the upstream had the lowest mean lead and cadmium concentrations (0 mg L^−1^), and this was the same as control (*p* ≤ 0.05) and significantly lower than NESREA standard limit (0.01 and 0.005 mg L^−1^), respectively.

**Table 2:** Mean Concentration of lead (Pb), cadmium (Cd), and iron (Fe) in the Ori‐Ile stream water samples. (Means with the same letter (for each month) are not significantly different at *p* ≤ 0.05. WHO‐L: WHO limit; NESREA‐L: NESREA limit; SD: Standard deviation)

Heavy metals	Sampling points	March 2008	May 2008	July 2008	September 2008	January 2009	March 2009	May 2009	July 2009	Overall mean
Pb [mg L^−1^]	Downstream	0a)	0a)	0a)	0a)	0a)	0a)	2.1500 ± 0.07a)	0.0550 ± 0.007a)	0.275 ± 0.0096
	Midstream	0a)	0a)	0a)	0a)	0a)	0a)	0.1550 ± 0.007b)	0.0350 ± 0.007b)	0.0238 ± 0.0018
	Upstream	0a)	0a)	0a)	0a)	0a)	0a)	0c)	0c)	0.0000
	WHO‐L	0.0100b)	0.0100b)	0.0100a), b)	0.0100b)	0.0100a), b)	0.0100c)	0.0100a)	0.0100a)	0.0100
	NESREA‐L	0.0500	0.0500	0.0500	0.0500	0.0500	0.0500	0.0500	0.0500	0.0500
	Control	0.0000a)	0.0000a)	0.0000a)	0.0000a)	0.0000a)	0.0000a)	0.0000c)	0.0000c)	0.0000
	Mean	0	0	0	0	0	0	0.7683	0.0300	0.0998
	SD	0	0	0	0	0	0	1.0729	0.0253	0.1373
	Mean^2^	0.0000^nd^	0.0000^nd^	0.0000^nd^	0.0000^nd^	0.0000^nd^	0.0000^nd^	2.8755*	0.0016*	–
Cd [mg L^−1^]	Downstream	0b)	0b)	0.0075 ± 0.002a)	0.0100a)	0.0065 ± 0.0007a)	0b)	0.1710 ± 0.004a)	0.0065 ± 0.0007a)	0.0252 ± 0.0009
	Midstream	0b)	0b)	0b)	0.0070 ± 0.001b)	0.0055 ± 0.0007a)	0b)	0c)	0.0050 ± 0.001a)	0.0022 ± 0.0003
	Upstream	0b)	0b)	0b)	0c)	0b)	0b)	0c)	0b)	0.0000
	WHO‐L	0.0030a)	0.0030a)	0.0030a), b)	0.0030a), b)	0.0030a), b)	0.0030a)	0.0030b)	0.0030a), b)	0.0030
	NESREA‐L	0.0100	0.0100	0.0100	0.0100	0.0100	0.0100	0.0100	0.0100	0.0100
	Control	0.0000b)	0.0000b)	0.0000b)	0.0000c)	0.0000b)	0.0000b)	0.0000c)	0.0000b)	0.0000
	Mean	0	0	0.0025	0.0057	0.0040	0	0.0570	0.0038	0.0091
	SD	0	0	0.0040	0.0046	0.0032	0	0.0883	0.0031	0.0129
	Mean^2^	0.0000^nd^	0.0000^nd^	0.000038*	0.000053*	0.000025*	0.0000^nd^	0.0195*	0.000023*	–
Fe [mg L^−1^]	Downstream	0.100 ± 0.014b)	12.900 ± 0.07a)	0.450 ± 0.007b)	0.300b)	0.750 ± 0.007b)	0.400 ± 0.014b)	0.650 ± 0.007b)	0.750 ± 0.007b)	2.0375 ± 0.0158
	Midstream	0.0100 ± 0.014b)	0b)	0.350 ± 0.007b)	0.100c)	0.450 ± 0.007c)	0.0200b), c)	0.550 ± 0.007b)	0.350 ± 0.007c)	0.2625 ± 0.0053
	Upstream	0b)	0b)	0.250 ± 0.007b)	0d)	0d)	0c)	0.550 ± 0.007b)	0.200c)	0.125 ± 0.0018
	WHO‐L	0.3000a)	0.3000a), b)	0.3000a)	0.3000a)	0.3000a)	0.3000a)	0.3000a)	0.3000a)	0.3
	NESREA‐L	1.0000	1.0000	1.0000	1.0000	1.0000	1.0000	1.0000	1.0000	1.0000
	Control	0.0163b)	0.0163b)	0.0163b), c)	0.0163b), c)	0.0163c), d)	0.0163b), c)	0.0163c)	0.0163c)	0.0163
	Mean	0.0067	0.4300	0.0350	0.0133	0.0400	0.0200	0.0583	0.0433	0.080825
	SD	0.0103	0.6669	0.0105	0.0137	0.0341	0.0190	0.0075	0.0258	0.098475
	Mean^2^	0.000067	1.1094*	0.0002	0.00047*	0.0029*	0.0008*	0.000067	0.0016*	–

^a)^ Means with the same letter (for each month) are not significantly different at p ≤ 0.05

^b)^ WHO‐L: WHO limit

^c)^ NESREA‐L: NESREA limit

^d)^ SD: Standard deviation; nd = not detected (values less than 0.001 were not detected during AAS analysis); Analysis of variance (Mean^2^ values) with * are significant at p ≤ 0.05.

The results obtained showed that iron concentration in the upstream ranged from 0.000 mg L^−1^ (March–May 2008, September 2008, and January–March 2009) to 0.055 (May 2009) with a mean and standard deviation of 0.125 ± 0.0018 mg L^−1^. The midstream iron concentration ranged from 0.000 mg L^−1^ (May 2008) to 0.055 (May 2009) with a mean and standard deviation of 0.263 ± 0.0053 mg L^−1^. The downstream iron concentration ranged from 0.100 mg L^−1^ (March 2008) to 1.290 mg L^−1^ (May 2008) with a mean and standard deviation of 0.586 ± 0.016 mg L^−1^. The results obtained showed that iron concentration in the control stream was 0.0163 mg L^−1^. Overall, downstream had the highest mean iron concentration (0.586 mg L^−1^), which was significantly higher than control (*p* ≤ 0.05) but less than the NESREA standard limit (1.0 mg L^−1^), while the upstream had the lowest mean iron concentration (0.125 mg L^−1^) and this was significantly lower than control (*p* ≤ 0.05) and NESREA standard limit (0.005 mg L^−1^). The analysis of variance (ANOVA) results showed that the differences in mean lead concentration in the surface water samples were significant at *p* = 0.05 only in May 2009 and July 2009 while that of cadmium and iron concentrations in the surface water samples were significant at *p* ≤ 0.05 except in March 2008, May 2008, and March 2009 for cadmium, and in March 2008 and May 2008 for iron.

The results of the studied concentration of lead and cadmium in Omilende stream water showed that downstream had the highest value followed by the midstream. However, lead and cadmium were not detected in the upstream location of the study and the control sites. The values obtained for the concentrations of lead and cadmium in the downstream were very much higher than the NESREA standard but those in the midstream were less than NESREA standard. This result is in agreement with the observation of Adeagbo^41^ that the concentrations of lead and cadmium exceeded the maximum permissible level in surface waters of Olodo, Ikumapaiyi, and Arubiewe. Leachate from the waste batteries and accumulators' dumpsites that came in contact with surface water was considered to be responsible for the high values of the metals. The location of the midstream at the base of the waste dumpsite and the downstream away from the dumpsite following the direction of the water flow makes the pattern observed in the results of this study similar to that obtained by Adeagbo.^41^ One of the greatest problems of water supply and usage, especially in the developing areas such as Omilende Olodo, is the condition of the water. Often times, water runoff, a nonpoint source of pollution, carries toxic pollutants from land areas into streams and rivers, thus causing serious aquatic pollution. Similarly, according to WHO,^42^ contamination of drinking water is a significant concern for public health throughout the world because chemicals in water supplies can cause serious health problems, whether the chemicals are naturally occurring or derived from sources of pollution. Thus, lead and cadmium, along with other heavy metals in the wastes, were eroded from the topsoil by rain water and were deposited into the stream. This is of considerable health and environmental concerns due to their toxicity and bioaccumulative behavior.^43^, ^44^, ^45^ According to Blacksmith Institute,^46^ this polluted source of water supplies when used for domestic and agricultural purposes could serve as a route through which these toxic metals enter into the food chain. The general population may get exposed to lead and cadmium, through the consumption of the contaminated Omi stream water, and this could result in acute toxicity over time through the process of bioaccumulation. Additionally, the exposure of Ori‐Ile residents could occur through the consumption of food grown in gardens that were irrigated with the polluted Omi stream water, and this may eventually lead to serious health challenges in these residents.

### 2.3 Pb, Cd, and Fe Concentrations in Maize Parts

The concentrations of Pb, Cd, and Fe measured in the different parts of the cultivated maize namely roots, stems, leaves, and grains were compared to values obtained from reference site maize.

*Pb Concentrations*: The result obtained showed a significant difference among the mean Pb concentration of the different maize parts, and Student's *t*‐test results showed that the differences in mean values for the different maize parts were significant at *p* ≤ 0.05. The cultivated maize root had the highest Pb concentration than the other maize parts.

*Cd Concentrations*: The result obtained showed a significant difference among the mean Cd concentration of the different maize parts. The Student's *t*‐test results showed that the differences in mean values for the different maize parts were significant at *p* ≤ 0.05. The cultivated maize root had the highest Cd concentration than the other maize parts.

*Fe Concentrations*: The result obtained showed a significant difference among the mean Fe concentration of the different maize parts. The Student's *t*‐test results showed that the differences in mean values for the different maize parts were significant at *p* ≤ 0.05. The cultivated maize leaf had the highest Fe concentration than the other maize parts.

**Figure 4:**
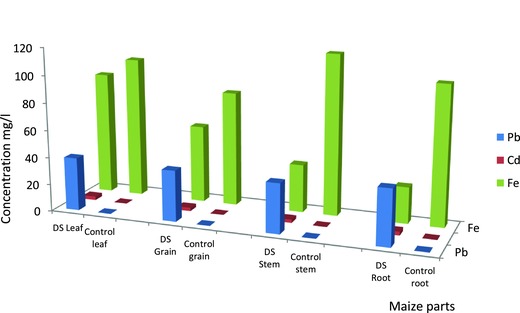
Lead, cadmium, and iron in cultivated maize parts and control. Key: Each bar represent maize parts; successive colors represents parts' heavy metal concentrations; DS, dumpsite area; Control: reference soil.

From the results obtained, the TF in all maize‐parts was less than 1 for all the three heavy metals. However, the maize root had the highest TF (TF_root_ > TF_leaf_ > TF_grain_ > TF_stem_) (**Figure** **4** and **Table** **3**). Only the PLI values for Cd were above 1, which indicated that there was higher uptake and accumulation of Cd in the maize plant from the polluted soil of the garden around the waste dumpsite while Pb and Fe levels were at the baseline.

**Table 3:** Transfer factors and pollution load indices for lead, cadmium, and iron in the cultivated maize parts. (TF/PLI < 1 = low; TF/PLI > 1 = high)

Calculated indices	Maize parts	Pb	Cd	Fe
Transfer factor (TF)	TF maize grains	0.0096	0.1803	0.0048
	TF maize stem	0.0094	0.1755	0.0046
	TF maize root	0.0105	0.1973	0.0052
	TF maize leaf	0.0100	0.1881	0.0050
Pollution load index (PLI)	PLI maize grains	0.668	1.766	0.530
	PLI maize stem	0.663	1.760	0.523
	PLI maize root	0.688	1.830	0.545
	PLI maize leaf	0.677	1.801	0.537

Plants have evolved detoxification mechanisms,^47^ and the efficiency of these processes might result in the tolerance of the natural heavy metals.^48^ Cereals such as maize are known to be good accumulators of contaminants.^29^, ^30^ Lead and cadmium are the most widespread no‐nutrient heavy metals.^31^ Results obtained in this study showed high concentrations of Pb, Cd, and Fe in the roots, stems, leaves, and grains of the maize plants when compared with levels in the same parts in reference site samples. The concentrations of Pb, Cd, and Fe found in the Ori‐Ile maize grain were considerably high, but the Pb and Cd levels in the Ori‐Ile maize root were the highest. According to Goldsbrough et al.,^49^ uptake of toxic metals in plants along with vital elements occurs primarily through the root system. This corroborates the results obtain from the maize root and further clarifies why the concentration found in the root of the Ori‐Ile maize was significantly high.

The highest concentration of Fe was found in the Ori‐Ile maize leaf. According to Aliu et al.,^30^ maize leaf is an active site for photosynthetic activities and the primary location of food production. This explains why Fe concentration in the leaf area was high since Fe is essentially required for photosynthesis. Fe is an essential plant nutrient and it functions to accept and donate electrons; it also plays important roles in the electron‐transport chains of photosynthesis and respiration.^50^ However, Fe is toxic when it accumulates to high levels and can act catalytically via the Fenton reaction to generate hydroxyl radicals, which can damage lipids, proteins, and DNA.^50^

High levels of Pb, Cd, and Fe in Ori‐Ile maize parts could only be attributed to contamination of the soil on which the maize plants were grown. Cunningham and Ow^22^ stated that plants can thrive in soil contaminated to levels that are often orders of magnitude higher than the standard regulatory limits. Olusoga and Osibanjo^23^ obtained similar Pb and Cd concentrations in their study on metals in plant species found in the dumpsite at Olodo area (Pb (32.6–295.3 mg Pb kg^−1^ dry weight), Cd (0.93–56.14 mg Cd kg^−1^ dry weight)) and those found in farmland (Pb (31.8–88.75 mg Pb kg^−1^ dry weight), Cd (Not Detected (N.D). to 29.40 mg Cd kg^−1^ dry weight)). Their results showed that the concentrations exceeded normal range of Pb and Cd in plants, though, the range they reported fall within critical concentrations of these two metals in plants.^51^, ^52^, ^53^ The results obtained in this study are compared well with the results reported by Olusoga and Osibanjo^23^ for Cd while Pb level in this study was slightly higher.

Heavy metals like Pb and Cd have been shown to affect plant growth and production in a multiple way especially by inhibiting a number of physiological processes in plants.^30^ Wallace et al.^54^ and Barcelo et al.^55^ reported that Pb and Cd are among the heavy metals that cause disturbance in plants' ion balance as well as in plants' water balance. They further stated that this could eventually result in interference with protein metabolism by influencing nitrate and sulfate reduction.^56^, ^57^ Pb has also being said to inhibit chlorophyll synthesis by causing impaired uptake of essential elements by plants,^58^ and high Pb content could lead to reduced vascular tissues.^59^ Therefore, the uptake of Pb by the Ori Ile maize plant could be detrimental as it hinders the uptake of the essential mineral elements needed by this plant. Aliu et al.^30^ studied the exposure of maize seedlings to Pb, Cd, and Hg, and observed that the leaf area had the highest values of the heavy metals with a value of 82.01%. Though the result obtained in this study indicated that the root had the highest concentration for lead and cadmium, the concentration found in the leaf was also significantly high.

Malecka et al.^60^ indicated that Pb concentration in the exposed plant increased as its concentration in the soil increased. However, the results of this study affirmed maize as a significant accumulator for Pb, Cd, and Fe. Results obtained in this study likewise showed that the concentration of Cd is relatively low compared to the concentration of Pb. Fe concentration was found significantly less than the level of Fe in the reference site. According to Spectrum Analytic,^20^ toxicity of Fe is primarily pH related and its visual symptoms are likely to be the deficiency of other nutrient(s). The pH value obtained in soils of Ori‐Ile dumpsite environment was acidic (pH < 6) and it could be due to the fact that the maize plants had easy access to Fe and as such absorbed significant concentration from the soil.

## 3 Conclusion

The dispersal of the heavy metal components of wastes to other parts of the community was also confirmed by the concentrations found in surface water. The result obtained showed significant concentration of the metals in the water body. This further showed that these metals were gradually washed via erosion or leached into the water sources from the soil which had high concentration of the metals. This calls for urgent attention because lead and cadmium are bioaccumulative in nature, and their continual exposure through the water sources could be detrimental to the residents' health as well as to their animals.

Furthermore, heavy metal accumulation in soils and plants is of growing concern due to the potential risks to human health.^30^, ^61^ This eventually often leads to food chain contamination which is one of the important pathways for the entry of these toxic pollutants into the human body.^62^, ^63^ Therefore, according to Muchuweti et al.,^11^ the extreme buildup of heavy metals in soils can lead to higher heavy metal uptake by plants, and this can affect the quality and safety of food. Consequently, as the analyzed heavy metals' concentration in the most consumed parts of the experimental maize was high, there is a tendency of these metals being transferred to both animals and humans who consume contaminated maize parts regularly. This may result in several unpleasant health effects, if bioaccumulated. Thus, while the remediation of the polluted soils of Ori‐Ile battery waste dumpsite is highly recommended, the garden maize is unfit for consumption.

## 4 Experimental Section

**Figure 5:**
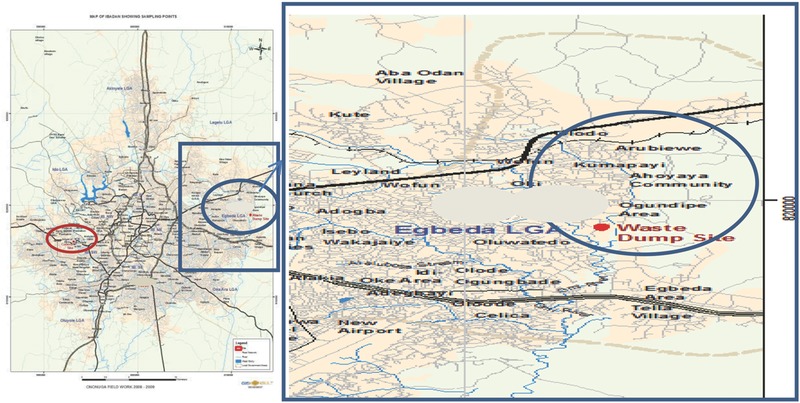
Map of Ibadan showing A) the Ori‐Ile waste dumpsite and B) reference site. (Source: GIS Konsult, Bodija, Ibadan).

**Figure 6:**
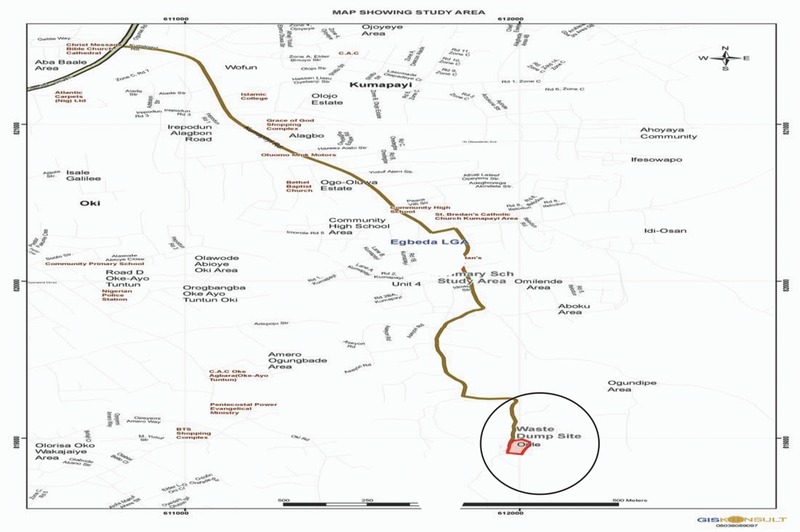
Map of Olodo showing the Ori‐Ile waste dumpsite (Source: GIS Konsult, Bodija, Ibadan).

*Study Area*: The study area was a semiurban residential and agricultural area located on latitudes 7°24′28.1″N, longitudes 4°00′52.2″E, and elevation 176 m, respectively. It contained a waste dumpsite located at Omilende village in Ikumapaiyi Area of Olodo community, northwest of Egbeda Local Government Area, Ibadan, Oyo‐State. It is popularly referred to as Ori‐Ile Waste Dumpsite. **Figures** **5** and **6** show the map of Ibadan with the location of the study site and the map of the study area (Olodo). The study site was a large and bare expanse of land of about 2 hectares, characterized with scanty vegetation. The most abundant groups of vegetation on and around the waste dumpsite were grasses, some of which included *Panicum clandestinum* (corn grass), *Muhlenbergia emersleyi* (bull grass), and *Echinopogon ovatus* (hedgehog grass). *P. clandestinum* was most abundant in areas surrounding the study site. The area has a bimodal rainfall pattern which peaks in June and September. The site was used as an unapproved waste dumpsite for battery wastes from the now closed‐down company called West African Battery Industry, who used to produce the battery known as “Exide Battery.” It was also used as an informal lead recovery site by informal and local Used Lead Acid Battery operators.

The area surrounding the waste dumpsite is inhabited by people who are mostly peasant farmers and traders. Some of the residents have gardens in their yard. Among the plants found in these gardens were *Z. mays*, *Carica papaya* (Pawpaw), *Lycopersicon esculentum* (Cocoyam), *Discorea sp* (Yam), etc. Domestic animals are raised by residents living around the study area. Some of them are *Gallus gallus domesticus* (both local and broiler chicken) and *Capra aegagrus hircus* (Nigerian Dwarf Dairy Goat). The reference site was located at National Center for Genetic Resources and Biotechnology, Moor Plantation, Ibadan, latitude 7°23′31.5″N and longitude 3°50′46.5″E.

*Sampling Procedure and Analysis—Soil*: Forty top soil samples were obtained from the study site in 2008 (March, May, July, and September) and 2009 (January, March, May, and July). The guidelines provided by US Environmental Protection Agency^64^ were employed during sampling. The soil samples were taken at the top region only (0–15 cm deep); along a straight line from A to B with midpoint M and bulked together to form a composite sample. Geographical Positioning System (GPS) was employed in acquiring location information on specific points of sample collection during each visit (**Table** **4**).

**Table 4:** GPS data of the soil sampling points of waste dumpsite and reference site

Sampling area	Sampling distance [m]	No. of samples per visit	Sampling points	GPS coordinates		
				Elevation [m]	North	East
Experimental garden direction (E)	0	One composite sample (E0)	AE 0	174	07°24′29.3″	004°00′52.6″
			ME 0	172	07°24′29.4″	004°00′53.5″
			BE 0	171	07°24′29.2″	004°00′54.4″
	10	One composite sample (E10)	AE 10	174	07°24′29.7″	004°00′52.6″
			ME 10	171	07°24′29.6″	004°00′53.6″
			BE 10	172	07°24′29.5″	004°00′54.5″
	20	One composite sample (E20)	AE 20	171	07°24′30.0″	004°00′52.6″
			ME 20	166	07°24′29.9″	004°00′53.8″
			BE 20	170	07°24′29.7″	004°00′54.4″
	25	One composite sample (E25)	AE 25	172	07°24′30.2″	004°00′52.8″
			ME 25	170	07°24′30.1″	004°00′53.8″
			BE 25	169	07°24′29.9″	004°00′54.5″
Waste dumpsite sample	Random	One composite sample (Rd)	Rd 1	173	07°24′28.6″	004°00′53.0″
			Rd 2	174	07°24′28.5″	004°00′53.8″
			Rd 3	175	07°24′26.8″	004°00′53.3″
			Rd 4	175	07°24′27.1″	004°00′52.6″
Reference site (C)	Random	One composite sample	C		07°23′31.5″	003°50′46.5″

The soil samples were collected from the waste dumpsite and along the direction of the experimental garden at 5 m intervals from its edge with the aid of a soil auger and a hand trowel in clean, well‐labeled polythene sampling bags. The sampling bags were sealed to prevent contamination during transportation to the laboratory and were then taken to the laboratory for processing and analysis. The digestion of the samples was carried out according to the method adopted by Adie and Osibanjo.^38^ Air‐dried soil samples (≈1 g each) were accurately weighed into a series of 100 mL beakers, and 10 mL of 2 m HNO_3_ was measured with a 10 mL pipette and added into each of the beakers (with watch glass covers) containing the soil samples. These beakers were shaken properly and placed on a water bath, which was boiled at 100 ± 30 °C, and these beakers with their contents were intermittently shaken after every 20 min. Acid‐extractable Pb, Cd, and Fe samples were leached for 2 h. The contents were filtered through Whatman's No.1 filter paper, and the filtrate of the digested samples was made up to a final volume of 100 mL by diluting it with deionized water depending on the suspected level of the metals. The metals, Pb, Cd, and Fe, were then analyzed by using a Perkin Elmer Analyst 200 Flame Atomic Absorption Spectrophotometer (2003 model). A reagent blank sample was taken through the method, analyzed, and subtracted from the samples to correct for reagent impurities and other sources of errors from the environment. The procedure was repeated for all the samples and their replicates. Reference soils were also collected and subjected to the same procedural analysis.

**Figure 7:**
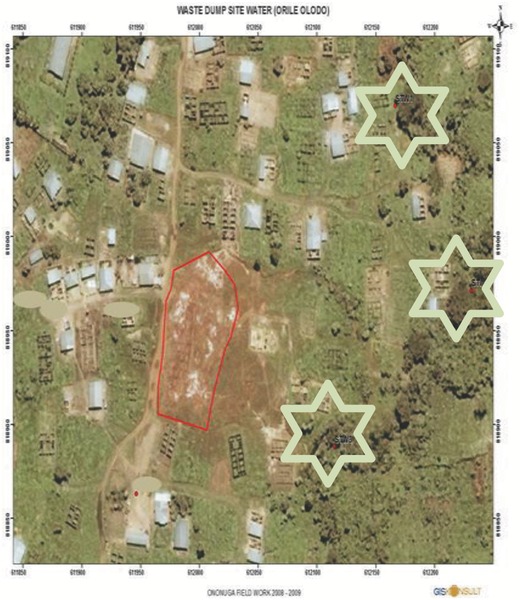
Satellite image of Omilende showing the Ori‐Ile waste dumpsite (red highlight) and surface water route (star highlight).

*Sampling Procedure and Analysis—Surface Water*: Surface water samples were randomly collected at three representative points along the stream (upstream, midstream, and downstream) (**Figure** **7**). The samples were collected in precleaned plastic bottles, and were labeled upstream water which was a point far off from where the waste dumpsite is located (latitude 07°24′31.7″N, longitude 004°00′59.0″E), midstream water which was the point perpendicular to the waste dumpsite location (latitude 07°24′28.5″N, longitude 004°01′01.1″E), and downstream water which was a point further down beyond the location of the waste dumpsite (latitude 07°24′25.8″N, longitude 004°00′57.3″E), respectively (Figure 7). Control stream water was taken from Moor plantation stream water (latitude 7°23′31.5″N and longitude 3°50′46.5″E) at National Centre for Genetic Resources and Biotechnology, Moor Plantation, Ibadan.

These samples were taken once every 2 months for 18 months. Few drops of HNO_3_ were added in order to prevent the loss of metals, and bacterial and fungal growth. Temperature and pH of water samples were also measured at the time of collection. The samples were taken to the laboratory and kept frozen in the refrigerator until analysis.

Filtered acidified samples (using nitric acid) were analysed for dissolved elements, and the whole samples were digested with nitric acid (ISO 15587‐2) to give the concentrations of Pb, Cd and Fe in the surface water samples using a Perkin Elmer Analyst 200 Atomic Absorption Spectrophotometer (2003 Model). The technique is based on the measurement of specific isotopes of these elements. Corrections were made for isobaric interferences and interferences by polyatomics. Correction factors and correction equations were evaluated during the run by analyzing a so‐called interference check solution.^66^

*Sampling Procedure and Analysis—Maize*: To determine the levels of uptake and accumulation of Pb, Cd, and Fe in plants, maize was planted in a nearby house garden tagged “experimental garden.” The garden was situated within 25 m away from the waste dumpsite. At maturity, the root, stem, leaf, and grains were harvested. Reference maize plants were also cultivated and the parts were also harvested at maturity. All harvested maize parts were kept in well‐labeled polythene bags and taken to the laboratory for further analysis. At the laboratory, each harvested maize part was washed with deionized water to remove any debris prior to drying. Representative amounts of the plant tissues were oven‐dried at 65 °C to constant weights and ground in a porcelain mortar. About 0.02–1 g of the ground samples was weighed in Vitreosil crucibles and dry‐ash in a muffle furnace at 450–500 °C for 3 h. The ash was dissolved in 5 mL of hot 6 m nitric acid and evaporated on a hotplate. After evaporation, 1 mL of 6 m nitric acid was added followed by deionized water, and then the sample solution was filtered. The filtered extract was poured into a volumetric flask, made up to 25 mL with deionized water and then stored in acid‐washed plastic vials with screw caps prior to analysis.[67 Extracts of the plant samples were then analyzed for lead, cadmium, and iron using a flame atomic absorption spectrophotometer (Buck 200A).

*Sampling Procedure and Analysis—Quality Control*: Quality control of metal analysis was performed by analyzing reference samples of both plants and soil, and for reagents the quality assurance scheme included blank reagents.

*Sampling Procedure and Analysis—Calculated Factors*: CF and TF were determined for soil and maize, respectively, using the methods adopted by Agunbiade and Fawale^68^ and by Cui et al.^69^

1. CF is often used to access soil contamination through the comparison of the concentrations in the surface layer to the background values. It is calculated using the equation
  (1) $$\mathrm{CF}=C_{\left(0-1\right)}/C_{n}$$

where CF is the contamination factor, *C*_(0–1)_ is the mean of concentrations of individual metal from all test sites, *C_n_* is the baseline or background concentration of individual concentration of metals at control site.
CF < 1: Low contamination factor,
1 < CF < 3: Moderate contamination factor,
3 < CF < 6: Considerable contamination factor,
CF > 6: Very high contamination factor.

2. TF is the concentration of each of the metal in the maize relative to the level of the metal in the corresponding soil on dry weight basis, respectively.
  (2) $$\mathrm{TF}=C_{p}/C_{s}$$

where *C*_p_ is the concentration of the metal in the plant (maize) while *C*_s_ is the concentration of the metal in the corresponding soil:^69^
TF > 1: High level of accumulation of metal in the plant.

*Statistical Analysis*: The results of all the heavy metals in the soil samples were grouped and analyzed using a factorial tool to compress the data and identify patterns of relationship within them. The results of the heavy metals in the maize parts were analyzed for level of significance using the Student's *t*‐test with a significance level of *p* ≤ 0.05.

## Conflict of Interest

The authors declare no conflict of interest.
